# Ambient Air Pollution and Daily Outpatient Visits for Cardiac Arrhythmia in Shanghai, China

**DOI:** 10.2188/jea.JE20140030

**Published:** 2014-07-05

**Authors:** Ang Zhao, Renjie Chen, Xingya Kuang, Haidong Kan

**Affiliations:** 1 School of Public Health, Key Lab of Public Health Safety of the Ministry of Education, Institute of Global Health, Fudan University, Shanghai, China; 2 Shanghai Key Laboratory of Atmospheric Particle Pollution and Prevention (LAP^3^), Fudan University, Shanghai, China; 3 Research Institute for the Changing Global Environment and Fudan Tyndall Centre, Fudan University, Shanghai, China; 4 Department of Occupational Medicine, Shanghai Yangpu District Central Hospital, Shanghai, China

**Keywords:** air pollution, outpatient visits, cardiac arrhythmias, time-series

## Abstract

**Background:**

Cardiac arrhythmias are cardiac rhythm disorders that comprise an important public health problem. Few prior studies have examined the association between ambient air pollution and arrhythmias in general populations in mainland China.

**Methods:**

We performed a time-series analysis to investigate the short-term association between air pollution (particulate matter with an aerodynamic diameter less than 10 µm [PM_10_], sulfur dioxide [SO_2_], and nitrogen dioxide [NO_2_]) and outpatient visits for arrhythmia in Shanghai, China. We applied the over-dispersed Poisson generalized additive model to analyze the associations after control for seasonality, day of the week, and weather conditions. We then stratified the analyses by age, gender, and season.

**Results:**

We identified a total of 56 940 outpatient visits for cardiac arrhythmia. A 10-µg/m^3^ increase in the present-day concentrations of PM_10_, SO_2_, and NO_2_ corresponded to increases of 0.56% (95% CI 0.42%, 0.70%), 2.07% (95% CI 1.49%, 2.64%), and 2.90% (95% CI 2.53%, 3.27%), respectively, in outpatient arrhythmia visits. The associations were stronger in older people (aged ≥65 years) and in females. This study provides the first evidence that ambient air pollution is significantly associated with increased risk of cardiac arrhythmia in mainland China.

**Conclusions:**

Our analyses provide evidence that the current air pollution levels have an adverse effect on cardiovascular health and strengthened the rationale for further limiting air pollution levels in the city.

## INTRODUCTION

Epidemiologic studies have demonstrated associations between short-term increases in outdoor air pollution concentrations and adverse cardiovascular effects, including increased mortality and hospital visits for ischemia heart disease, heart failure, and sudden cardiac arrest.^1^ The underlying mechanisms remain unclear, but the cardiac autonomic nervous system may be largely involved.^2^

Cardiac arrhythmias are cardiac rhythm disorders that comprise an important public health problem.^3^ Cardiac arrhythmias are associated with increased risk of cardiovascular complications and sudden cardiac arrest (or death), consequently leading to decreased quality of life, disability, and high mortality risk and healthcare expense.^3^ Dozens of recent studies have suggested that particulate matter and other air pollutants might be responsible for cardiac arrhythmias. However, most of these results were obtained from small groups of subjects treated by implantable cardioverter defibrillators (ICD) and dynamic Holter electrocardiograms.^4^ Few epidemiologic studies in this area have been based on more widely generalizable populations. Furthermore, research has been conducted concerning the effects of outdoor air pollution on cardiac arrhythmias in mainland China where the air pollution levels are much higher than those in developed countries.

Therefore, we conducted a time-series analysis to examine the association between ambient air pollution and outpatient visits for cardiac arrhythmia in a general population of Shanghai, China.

## METHODS

### Data

The daily number of outpatient visits for cardiac arrhythmia from January 1, 2010, to December 31, 2011 (730 days), was obtained from Shanghai Yangpu District Central Hospital, also known as the Yangpu Hospital of Tongji University. As a major hospital located in the Yangpu District, which covers an area of 60 km^2^, the hospital provides medical services for approximately 1.3 million residents in and around this district. In recent years, the hospital had approximately 1.5 million outpatient and emergency room visits per year. In the outpatient department, the physicians must enter medical record data for each patient into the computer system. The information recorded includes individual characteristics (such as gender, age, and residence) as well as any diagnoses for the current visit. The diagnosis of cardiac arrhythmias is given by clinical physicians based on electrocardiogram criteria. In this study, to ensure accuracy of cardiac arrhythmia diagnosis, a statistical physician blinded to concentrations of pollutants was responsible for summarizing each day’s confirmed outpatient visits for cardiac arrhythmia, which excluded follow-up visits. Further, we also excluded a few patients from other districts (according to their recorded residency) in order to minimize the measurement error of air pollution exposure. Data were analyzed at the aggregate level, and no individual records/information for patients were used, so the study had a waiver of informed consent. Additionally, since no participants were contacted, written informed consent was not given by participants.

Daily 24-h air pollution concentrations, including particulate matter with an aerodynamic diameter less than 10 µm (PM_10_), sulfur dioxide (SO_2_), and nitrogen dioxide (NO_2_), were obtained from one state-owned air quality monitoring station located in the Yangpu District almost 2 km away from this hospital. According to Chinese government rules, the location of state-owned stations should not be in the direct vicinity of traffic or industrial sources. Methods based on tapered element oscillating microbalance, ultraviolet fluorescence, and chemiluminescence were used for the measurement of PM_10_, SO_2_, and NO_2_, respectively. To calculate the 24-h mean concentrations of PM_10_, SO_2_, and NO_2_, at least 75% of the 1-h values must have been available on that particular day.

To allow for adjustment for the potential confounding effects of weather on incidence of arrhythmias, daily 24-h mean temperature and relative humidity were obtained from the database of the Shanghai Meteorological Bureau.

### Statistical analysis

We adopted time-series regression models to investigate the short-term associations between air pollution and outpatient arrhythmia visits.^5^ Specifically, we applied a generalized additive model (GAM) to analyze the data. Because daily outpatient visits typically followed an over-dispersed Poisson distribution, we used a quasi-Poisson regression in the GAM. Consistent with previous time-series studies, we determined the model specifications empirically. We incorporated several covariates in the GAM as follows: (1) a natural cubic smooth function of calendar time with 7 degrees of freedom (df) per year to exclude unmeasured long-term and seasonal trends; (2) natural smooth functions of the mean temperature (6 df) and the relative humidity (3 df) to control for the potential nonlinear confounding effects of weather conditions; and (3) an indicator variable for “day of the week” to adjust for the day-in-week variation of the outpatient visits. We checked the autocorrelations of residuals using partial auto-correlation functions (PACF) for the above basic model. Because most previous studies suggest that the detrimental effects of air pollution on arrhythmia were limited to the first 24 h,^4^ we introduced air pollutant concentrations at the concurrent day into the basic model.

We also explored the age, sex, and season-specific effects of air pollution on arrhythmias. We tested the statistical significance of differences between effect estimates of the strata of a potential effect modifier (eg the difference between females and males) by calculating the 95% confidence interval (CI) as $({\hat{Q}}_{1}-{\hat{Q}}_{2})\pm 1.96\sqrt{S{\hat{E}}_{1}^{2}+S{\hat{E}}_{2}^{2}}$, where ${\hat{Q}}_{1}$ and ${\hat{Q}}_{2}$ are the estimates for the two categories, and $S{\hat{E}}_{1}$ and $S{\hat{E}}_{2}$ are their respective standard errors. Regardless of significance, we also considered modification of effect by a factor of two or more to be important and worthy of attention.^6^ For example, we considered sex to be an important modifier if the between-sex differences of effect estimates were more than doubled.

To check the stability of our results, we used alternative lag days of 1, 2, and 02 (3-day moving average) in the models. As another sensitivity analysis, we fitted two-pollutant models after controlling for other pollutants.

The statistical tests were two-sided, and effects of *P* < 0.05 were considered statistically significant. All models were fitted using R software version 2.15.1 (The R Foundation for Statistical Computing, Vienna, Austria) with the “mgcv” package. Results are presented as the percentage of change in the number of daily outpatient arrhythmia visits per 10-µg/m^3^ increase in pollutant concentrations.

## RESULTS

Table 1 summarizes the basic descriptive statistics of the study population and exposures. From 2010 to 2011 (730 days), a total of 56 940 outpatient visits for cardiac arrhythmia were identified. The annual mean average pollutant concentrations were 81 µg/m^3^ for PM_10_, 29 µg/m^3^ for SO_2_, and 54 µg/m^3^ for NO_2_. The annual average temperature and humidity were 17°C and 68%, respectively, reflecting the subtropical climate in Shanghai. Generally, both the outpatient visits and the air pollution levels were highest in cool seasons (see Table 1 and eTable 1), and PM_10_, SO_2_, and NO_2_ had moderate positive correlation coefficients with each other and were negatively weakly correlated with temperature and humidity (see eTable 2).

**Table 1.** Summary of descriptive statistics

	Mean ± SD	Minimum	P25	Median	P75	Maximum
Outpatient visits for arrhythmia (*n*)	78 ± 53	1	39	75	110	315
Age (y)						
0–65	34 ± 30	0	12	26	50	236
65<	45 ± 36	0	18	39	63	246
Gender (*n*)						
Male	36 ± 30	0	14	30	51	223
Female	43 ± 36	0	16	36	60	225
Season (*n*)						
Cool^a^	81 ± 54	0	41	78	112	291
Warm^b^	77 ± 53	0	38	72	109	315
Air pollutants concentrations (24-h average, µg/m^3^)						
PM_10_	81 ± 63	10	44	64	98	600
SO_2_	29 ± 18	7	16	24	37	130
NO_2_	54 ± 23	11	37	51	67	182
Weather conditions (24-h average)						
Temperature (°C)	17 ± 9	−2	9	18	25	36
Humidity (%)	68 ± 13	23	60	69	78	95

Abbreviations: SD, standard deviation; P (25), 25th percentile; P (75), 75th percentile; PM_10_, particulate matter with an aerodynamic diameter less than 10 µm; SO_2_, Sulfur dioxide; NO_2_, Nitrogen dioxide.

^a^Cool season: from November to April.

^b^Warm season: from May to October.

After checking the PACF plots, we did not find any apparent autocorrelations of the model residual, suggesting that the basic model may be appropriate. Table 2 shows the effect estimates using different lag days. The estimates decreased heavily from lag day 0 to lag day 2. The concurrent day concentrations generated much larger estimates than those on lag day 1 or 2. The effect of PM_10_ concentration was limited within the concurrent day, but the effects of SO_2_ and NO_2_ concentrations remained significant at lag day 1. A 10-µg/m^3^ increase in the concurrent day concentrations of PM_10_, SO_2_, and NO_2_ corresponded to increases in outpatient arrhythmia visits of 0.56% (95% CI 0.42%, 0.70%), 2.07% (95% CI 1.49%, 2.64%), and 2.90% (95% CI 2.53%, 3.27%), respectively. Percent increase in number of daily outpatient visits for arrhythmia associated with per interquartile range increase in pollutant concentrations using different lag days is displayed in eTable 3.

**Table 2.** Percent increase in number of daily outpatient visits for arrhythmia associated with a 10-µg/m^3^ increase in pollutant concentrations using different lag days in single-pollutant models

Lag	PM_10_	SO_2_	NO_2_
0	0.56 (0.42, 0.70)	2.07 (1.49, 2.64)	2.90 (2.53, 3.27)
1	−0.11 (−0.25, 0.03)	0.53 (0.01, 1.07)	1.10 (0.72, 1.48)
2	−0.01 (−0.15, 0.13)	0.23 (−0.29, 0.75)	0.14 (−0.24, 0.51)
02	0.18 (0.01, 0.35)	1.27 (0.59, 1.95)	2.27 (1.80, 2.74)

Values are reported as means and 95% confidence intervals.

Abbreviations: Lag day 02, 3 day-moving average concentrations.

Table 3 shows analysis results stratified by age, gender, and season. We observed significantly stronger associations of air pollutants and outpatient visits for arrhythmias in people aged 65 years or older. The effect of NO_2_ and SO_2_ among females was significantly stronger than males. Air pollutant effects varied by season; the effect of PM_10_ was three times larger in cool periods, while the effect of SO_2_ was almost four times larger in warm periods.

**Table 3.** Age, gender and season-specific percent increase in number of daily outpatient visits for arrhythmia associated with a 10-µg/m^3^ increase in pollutant concentrations on the concurrent day (lag 0) in single-pollutant models

	PM_10_	NO_2_	SO_2_
Age group			
≤65 years	−0.05 (−0.27, 0.18)*	2.00 (1.43, 2.57)*	−1.11 (−2.23, 0.01)*
>65 years	0.95 (0.78, 1.13)*	3.52 (3.03, 4.00)*	4.15 (3.42, 4.89)*
Sex			
Male	0.43 (0.23, 0.64)	1.97 (1.42, 2.53)*	1.38 (0.52, 2.25)*
Female	0.67 (0.49, 0.86)	3.69 (3.20, 4.18)*	2.69 (1.92, 3.46)*
Season			
Cool	1.03 (0.84, 1.22)*	2.55 (2.06, 3.05)*	1.09 (0.42, 1.75)*
Warm	0.33 (0.10, 0.56)*	3.56 (2.95, 4.16)*	4.03 (2.68, 5.39)*

Values are reported as means and 95% confidence intervals.

*Significant between-group difference (*P* < 0.05).

Table 4 compares the results of the single-pollutant and two-pollutant models. The effect of PM_10_ decreased only slightly after controlling for SO_2_ but substantially and with loss of statistical significance after controlling for NO_2_. The effect of SO_2_ appeared to decrease after adjusting for the two other pollutants. The effect of NO_2_ remained robust even after adjustment for PM_10_ and SO_2_.

**Table 4.** Percent increase in number of daily outpatient visits for arrhythmia associated with a 10-µg/m^3^ increase in pollutant concentrations on the concurrent day (lag 0) using single and two-pollutant models

	Model	Mean (95% CI)
PM_10_	Adjusted for SO_2_	0.41 (0.25, 0.58)
	Adjusted for NO_2_	−0.05 (−0.23, 0.13)
SO_2_	Adjusted for NO_2_	−0.94 (−2.15, 0.27)
	Adjusted for PM_10_	1.17 (0.51, 1.83)
NO_2_	Adjusted for SO_2_	4.39 (3.86, 4.92)
	Adjusted for PM_10_	2.98 (2.52, 3.44)

Values are reported as means and 95% confidence intervals.

Figure depicts the unadjusted concentration-response relationships for PM_10_, SO_2_, and NO_2_ with outpatient visits for arrhythmia. The concentration-response relationships appeared to be linearly positive without any thresholds.

**Figure.**
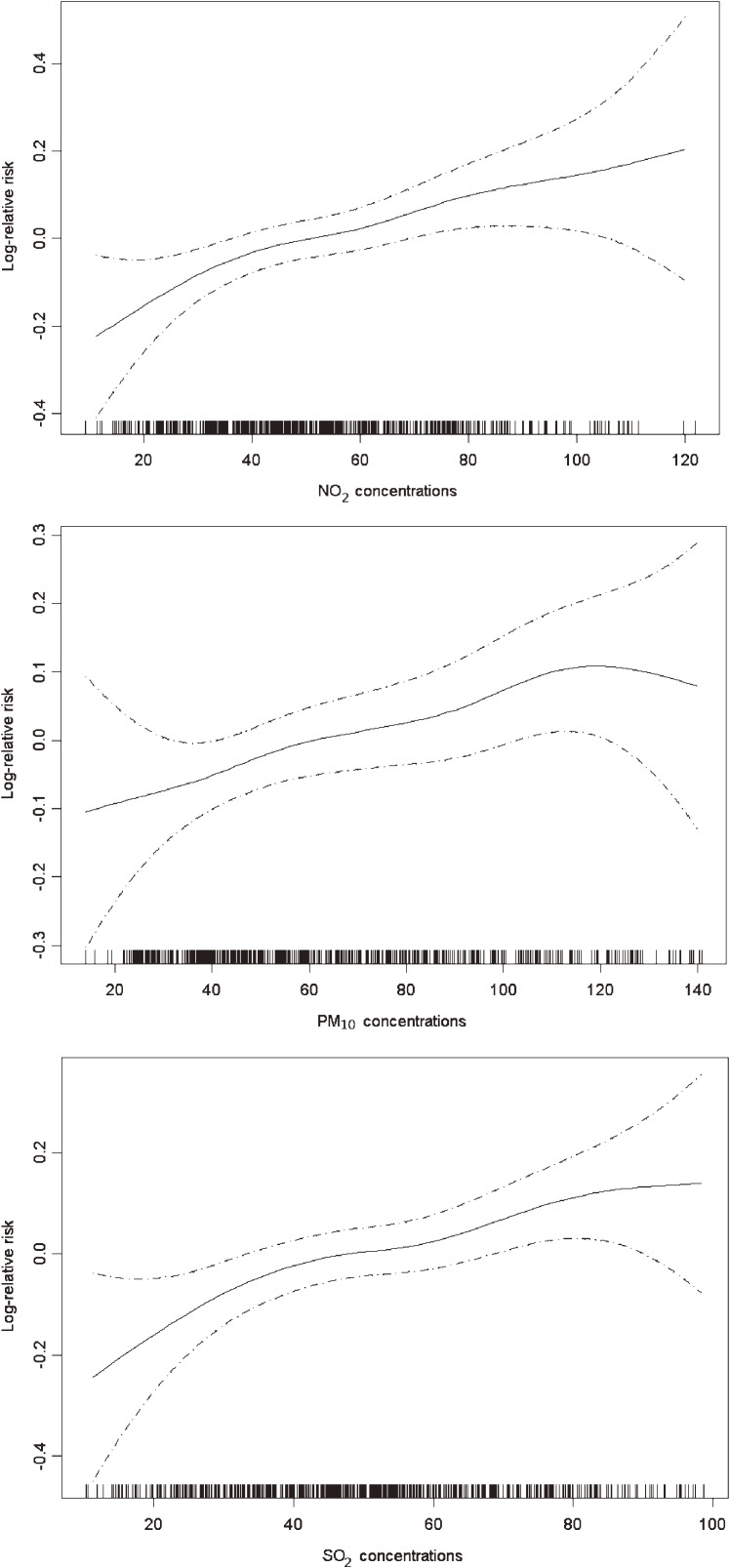
The concentration-response relationships of air pollutants and outpatient visits for arrhythmia. The X-axis is the pollutant concentrations (µg/m^3^) at the concurrent day. The Y-axis is the log-relative risk of outpatient visits for arrhythmia. The solid lines indicate the estimated mean change in the log-relative risk and the dotted lines represent the 95% confidence intervals of the estimates. The relative risk is expressed as the risk of outpatient visits for arrhythmia for certain concentrations of a pollutant compared to risk at its median concentration.

## DISCUSSION

In the present study, we found that increased concentrations of outdoor air pollutants were associated with increased outpatient visits for cardiac arrhythmia in a general population of Shanghai, China. These effects remained significant after using alternative lag selection and controlling for co-pollutants. To our knowledge, this is one of a small number of studies worldwide that have examined the association between air pollution and cardiac arrhythmias in general populations. Our findings could arouse public health concern about the adverse impact of air pollution on heart rhythms in China.

Our results are supported by those of previous epidemiologic studies. Most previous studies linking air pollution and arrhythmia were conducted in a small group of subjects who experienced repeated measures of heart rhythm events recorded using ICDs or Holter electrocardiograms, but the results were inconsistent.^4^ For example, Link et al found that PM_10_ was associated with increased risk of atrial fibrillation onset within hours following exposure in 49 patients with ICDs.^7^ Rich et al also suggested an increased risk of episodes of rapid ventricular response due to paroxysmal atrial fibrillation in the hour after exposure to ambient ozone.^8^ In several ICD-based studies, ventricular arrhythmia was also found to be triggered by air pollution exposure within 1 or 2 days.^9^^–^^11^ However, despite these findings, other studies have suggested insignificant associations between arrhythmia and air pollution.^12^^,^^13^ The Holter-based studies somewhat consistently reported significant adverse effects of air pollution within hours of exposure on ventricular repolarization,^14^ premature ventricular contractions,^15^ parameters of heart rate variation,^16^ and ST segment height.^17^

Our findings are also consistent with previous time-series or case-crossover studies using aggregate data, although this type of evidence was scarce. A time-series study in Sao Paulo, Brazil, found that concurrent-day concentrations of PM_10_, NO_2_, and carbon monoxide (CO) significantly influenced arrhythmia emergency room visits.^18^ Another time-series study in Helsinki, Finland, by Halonen et al also suggested a significant association between present-day PM_2.5_ and hospital admissions for arrhythmia.^19^ Two case-crossover studies in Taiwan found that PM_2.5_, NO_2_, CO, and ozone levels on lag day 0 were associated with increased numbers of emergency room visits for cardiac arrhythmia.^20^^,^^21^ Gerard et al found a significant association between air pollution and daily mortality caused by arrhythmia.^22^

Few studies examined age, sex, and season-specific effects of air pollution on arrhythmia. We found that the association between air pollution and arrhythmia in elderly participants aged 65 years or more was stronger than in younger subjects. It is plausible that older people have higher prevalence of chronic cardiopulmonary diseases and thus are more susceptible to the harmful effects of air pollution. The reasons for the observation of a larger effect in females than in males are complex and might be attributable to females’ relatively frail physique, greater deposition of air pollutants in the lungs higher airway responsiveness, and the generally unfavorable socioeconomic status of females in China.^23^^,^^24^ A number of previous air pollution epidemiologic studies have shown that females were slightly more sensitive to the detrimental effects of air pollution. Season is also an important factor that can modify the health effects of air pollution because of variations in air pollution content and levels and population exposure patterns.^24^ We found a stronger association between NO_2_ and PM_10_ and cardiac arrhythmias in the cool season but a larger effect of SO_2_ in the warm season. Further studies are needed to clarify this issue.

Our analyses indicate that gaseous pollutants, especially NO_2_, had larger and more robust effects on outpatient visits for arrhythmia than other pollutants, suggesting that more concern should be directed toward gaseous pollutants in terms of both future health studies and pollution control. The shapes of the concentration-response relationships are crucial for public health assessment, and there has been a growing demand for accurate curves. In the present study, we found that the relationship curves were almost linearly positive. Therefore, the current air quality standards (150 µg/m^3^, 150 µg/m^3^, and 80 µg/m^3^ for daily average concentrations of PM_10_, SO_2_, and NO_2_, respectively) might not be sufficient to ensure public health protection in Shanghai, and further control of air pollution is likely to improve health benefits.

Our study has two main strengths. First, our study is the first to explore the link between air pollutants and cardiac arrhythmias in China, where air pollution levels are very high. Second, compared to most previous studies in this area that focused on a small, select population of patients, our study examined associations in a relatively large general population in Shanghai.

Nevertheless, limitations should also be addressed. As in most previous time-series studies, we utilized measurements on a fixed-site monitoring station as a proxy for the population exposure level to air pollution. This might raise a number of issues, given that the ambient monitoring results may differ from the level of personal exposure to air pollutants, as people typically spend most of their time indoors.^25^ However, this measurement error would generally tend to bias estimates toward the null.^26^ We selected a central site for air quality monitoring in the same district as the hospital so we believe this measurement error may not be very large. Second, our health data were collected from only a single hospital, which may not represent the situation in the whole city. We were not able to obtain classification data for arrhythmia, thus limiting our ability to identify subgroups susceptible to air pollution exposure. Therefore, further studies on the association between air pollution and arrhythmia are needed.

## CONCLUSIONS

This time-series study found that air pollution might contribute to the increased number of outpatient visits for cardiac arrhythmia in Shanghai, China. Our results provide the first evidence that ambient air pollution is significantly associated with increased risks of cardiac arrhythmia in mainland China. Our findings might help local decision-makers establish air pollution control measures. Further large-scale studies are needed to confirm our results.

## ONLINE ONLY MATERIALS

eTable 1. Summary statistics (mean and standard deviation) of air pollutant concentrations and weather conditions in this study.

eTable 2. Pearson correlation coefficients between daily air pollutant concentrations and weather conditions in Metropolitan Shanghai (2010–2011).

eTable 3. Percent increase (mean and 95% confidence intervals) in number of daily outpatient visits for arrhythmia associated with an interquartile range increase in pollutant concentrations using different lag days in single-pollutant models.
