# Supplementary material for: Ambient Air Pollution and Daily Outpatient Visits for Cardiac Arrhythmia in Shanghai, China
Source: J Epidemiol. 2014 Jul 5;24(4):321–6. doi: 10.2188/jea.JE20140030 (PMC4074637; doi:10.2188/jea.JE20140030)
Supplement: eTable 1. [file je-24-321-s001.pdf]

eTable 1. Summary statistics of air pollutant concentrations and weather conditions

	PM_{10} (μg/m^3)	SO_2 (μg/m^3)	NO_2 (μg/m^3)	Temperature (°C)	Humidity (%)
Total	81±63	29±18	54±23	17±9	68±13
Spring	96±83	30±15	56±20	15±6	64±16
Summer	61±35	19±9	43±19	28±3	74±9
Autumn	74±62	24±14	54±26	20±5	70±11
Winter	92±54	44±22	63±25	6±4	66±13

Values are reported as mean±standard deviation

Spring: from March to May

Summer: from June to August

Autumn: from September to November

Winter: from December to February
