# Supplementary material for: Ambient Air Pollution and Daily Outpatient Visits for Cardiac Arrhythmia in Shanghai, China
Source: J Epidemiol. 2014 Jul 5;24(4):321–6. doi: 10.2188/jea.JE20140030 (PMC4074637; doi:10.2188/jea.JE20140030)
Supplement: eTable 2. [file je-24-321-s002.pdf]

eTable 2. Pearson correlation coefficients between daily air pollutant concentrations and weather conditions in Metropolitan Shanghai (2010-2011).

	SO_2	NO_2	Temperature	Humidity
PM_{10}	0.54	0.57	-0.18	-0.33
SO_2		0.71	-0.54	-0.40
NO_2			-0.33	-0.19
Temperature				0.23
