# Supplementary material for: Ambient Air Pollution and Daily Outpatient Visits for Cardiac Arrhythmia in Shanghai, China
Source: J Epidemiol. 2014 Jul 5;24(4):321–6. doi: 10.2188/jea.JE20140030 (PMC4074637; doi:10.2188/jea.JE20140030)
Supplement: eTable 3. [file je-24-321-s003.pdf]

eTable 3. Percent increase in number of daily outpatient visits for arrhythmia associated with an interquartile range increase* of pollutant concentrations using different lag days in single-pollutant models

Lag	PM_{10}	SO_2	NO_2
0	3.03 (2.28,3.77)	4.34 (3.13,5.54)	8.81 (7.70,9.93)
1	-0.58 (-1.35,0.19)	1.12 (-0.01,2.25)	3.34 (2.18,4.51)
2	-0.05(-0.79,0.68)	0.48 (-0.61,1.57)	0.42(-0.72,1.56)
02ł	0.95 (0.01,1.89)	2.66(1.23,4.09)	6.91(5.48,8.33)

Values are reported as means and 95% confidence intervals.

* The interquartile range concentrations of PM_{10}, SO_2, and NO_2 were 54 μg/m^3, 21 μg/m^3, and 30 μg/m^3, respectively.

ł Lag day 02 refers to the 3-day-moving average concentrations.
